# *Legionella bozemanii* lymphadenitis in an immunocompetent child and the role of sequencing in the diagnostic evaluation of culture-negative pediatric lymphadenitis: a case report

**DOI:** 10.1128/asmcr.00071-24

**Published:** 2025-07-31

**Authors:** Grace St. Cyr, Robert F. Potter, Katia C. Halabi, Coralee Del Valle Mojica

**Affiliations:** 1 Department of Pediatrics, Division of Infectious Diseases, Children’s Hospital of Philadelphia, Philadelphia, Pennsylvania, USA; 2 Department of Pathology and Laboratory Medicine, Perelman School of Medicine, University of Pennsylvania, Philadelphia, Pennsylvania, USA; 3 Department of Pathology and Laboratory Medicine, Children’s Hospital of Philadelphia, Philadelphia, Pennsylvania, USA; 4 Center for Microbial Medicine, Children’s Hospital of Philadelphia, Philadelphia, Pennsylvania, USA; 5 Infectious Diseases Division, Department of Pediatrics, Atlantic Health System, Goryeb Children’s Hospital, Morristown, New Jersey, USA; 6 Sidney Kimmel Medical College of Thomas Jefferson University, Philadelphia, Pennsylvania, USA; Pattern Bioscience, Austin, Texas, USA

**Keywords:** *Legionella bozemanii*, immunocompetent host, culture-negative lymphadenitis, 16s RNA sequencing, case report

## Abstract

**Background:**

*Legionella* species comprise a group of fastidious, intracellular, Gram-negative pathogens which are most associated with pulmonary disease, though extrapulmonary manifestations have been described. Some species, such as *Legionella bozemanii*, are thought to primarily affect immunocompromised hosts; however, prevalence may be underappreciated due to challenges in traditional culture-based detection methods.

**Case Summary:**

This case report describes a 3-year-old healthy male who experienced subacute cervical lymphadenitis refractory to medical and surgical management. After initial debridement, he had no growth on aerobic, anaerobic, acid-fast bacilli, or fungal cultures, at which time 16s rRNA sequencing was sent from lymph node aspirate. Following discharge testing resulted positive for *L. bozemanii,* and he responded well to 1 month of oral azithromycin therapy.

**Conclusion:**

To our knowledge, this is the first documented pediatric case of *L. bozemanii* both as a cause of cervical lymphadenitis and as a pathogen in an immunocompetent child. This case highlights the usefulness of molecular diagnostic methods in cases of refractory, culture-negative pediatric lymphadenitis and encourages increased diagnostic suspicion of *Legionella* species as causative agents of this disease.

## INTRODUCTION

*Legionella* species comprise a group of fastidious, intracellular, Gram-negative pathogens traditionally acquired through inhalation of aerosolized water particles from sources such as air-conditioning units or humidifiers (1). With over 60 species of *Legionella* identified, more than 30 of these are known to cause human disease. *Legionella pneumophila* is the primary pathogenic species implicated in syndromes such as pneumonia (Legionnaire’s disease) or febrile illness (Pontiac fever) (2). Some species, such as *Legionella bozemanii,* almost exclusively cause disease in immunocompromised hosts with increased risk of extrapulmonary manifestations, including skin and soft tissue pathology (3). However, extrapulmonary *Legionella* species infections in immunocompetent children are exceedingly rare, leading to challenges both in clinical suspicion and diagnosis due to lack of growth in routine culture media. Here, we present the first known case of an immunocompetent child with cervical lymphadenitis due to *L. bozemanii* diagnosed via 16S rRNA sequencing performed on lymphatic tissue. This case highlights the usefulness of molecular diagnostic methods in cases of refractory, culture-negative pediatric lymphadenitis and encourages increased diagnostic suspicion of *Legionella* species as causative agents of this disease.

## CASE PRESENTATION

A 3-year-old healthy male was transferred to our academic medical center for evaluation of subacute lymphadenitis refractory to medical and surgical management. Symptoms began 1 month prior with right cervical and submandibular swelling that developed over the course of 3 days and was associated with restricted neck range of motion. There was no known trauma to the area, dental procedures, or preceding viral illnesses. Detailed exposure history did not implicate any unusual source of infection; he lived in an apartment complex with his mother in New Jersey, often played in the soil outside and swam in the community pool but had no travel history outside of his county or other exposure to aquatic environments. He was treated with a 10-day course of oral amoxicillin 45 mg/kg/day divided twice daily without improvement and subsequently developed new fever. This led to a clinical diagnosis of right cervical lymphadenitis, which was treated with oral clindamycin 10 mg/kg/dose every 8 h with a plan for 7 days of therapy. However, symptoms persisted and on day 5 of antibiotics he was admitted to the referring facility for operative drainage of the cervical lymph node.

On admission he received intravenous (IV) ceftriaxone 75 mg/kg/dose once daily and IV clindamycin 13 mg/kg/dose every 8 h, promptly undergoing incision and drainage of the cervical lymph node. Surgical pathology demonstrated inflamed granulation tissue and fibrinopurulent exudate consistent with abscess. No special stains were performed. After several days of no growth from aerobic, anaerobic, and fungal cultures, he was transitioned to oral clindamycin 13 mg/kg/dose every 8 h to continue outpatient treatment. Within several days of discharge, he again had worsening of right cervical swelling and restricted range of motion with ongoing drainage from his incision site. He was readmitted to the referring facility for IV clindamycin 13 mg/kg/dose every 8 h and further evaluation with computed tomography (CT) scan of the neck on hospital day 2, which revealed a 2-cm rim-enhancing lesion inferior to the right submandibular gland suggestive of developing abscess. Aspiration of this lesion via interventional radiology was sent for aerobic culture, anaerobic culture, acid-fast bacilli (AFB) culture, fungal culture, and 16S rRNA sequencing evaluation (the Bacterial PCR reflex NGS assay from the University of Washington Molecular Microbiology Laboratory). Serologic tests were negative for acute Epstein-Barr virus infection (capsid IgM negative 23.2 [ref <36.00], early IgG negative <5.00 [ref <9.00], capsid IgG positive 649.00 [ref <18.00], nuclear antigen positive 145.00 [ref <18.00], indicative of past infection), cytomegalovirus (IgM negative 9.26 [ref <30.00], IgG negative <0.2 [ref <0.6]), and *Bartonella henselae* (IgM negative <1:20 [ref <1:20], IgG negative <1:128 [ref <1:128]). Interferon gamma release assay was also negative for evidence of exposure to tuberculosis (TB1- NIL value −0.0060 [ref <0.35], TB2-NIL value 0.0270 [ref <0.35], mitogen-NIL value >10.0 [no reference range], NIL value 0.121 [no reference range]). Given the lack of improvement on post-operative day 3 and lack of growth on standard cultures, he was transferred to our facility for further evaluation.

**Fig 1:**
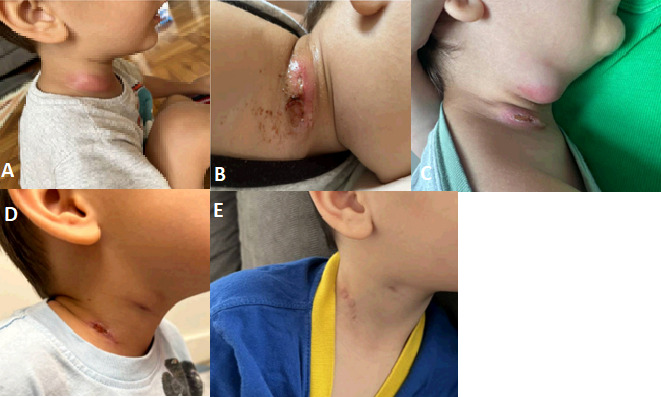
Progression of cervical and submandibular appearance over time. (**A**) Initial cervical swelling and erythema noted by the patient’s mother. (**B**) Drainage from the initial incision site, which prompted a return visit to the emergency department. (**C**) Progression of swelling despite drainage and antibiotic therapy noted on arrival to our facility. (**D**) Appearance at 1 month follow-up visit while on oral azithromycin with no recurrence of swelling or drainage noted. (**E**) Appearance at 2 months after completion of oral azithromycin therapy.

Within 24 h of admission to our facility, the patient underwent incision and curettage of the submandibular fluid collection and debridement of the lower cervical lymph node with significant purulence expressed from both cavities. Surgical pathology demonstrated granulomatous inflammation of both the submandibular and lower cervical lymph nodes. Grocott–Gömöri’s methenamine silver stain, acid-fast stain, Gram stain, and Fite stains were negative for bacterial or fungal elements on both samples. Given his reassuring clinical status and indolent nature of symptoms with highest suspicion for a nontuberculous mycobacterial infection, he was discharged home without antibiotics to await AFB culture and 16S rRNA sequencing testing sent from the prior aspiration. Following discharge, the 16S rRNA with reflex to next generation sequencing (NGS) testing resulted positive for *L. bozemanii,* and AFB culture was ultimately finalized with no growth. Given that the University of Washington sequencing assay is Clinical Laboratory Improvement Amendments (CLIA) validated and the results demonstrated only one pathogenic organism without other common water organisms, there was no concern for contamination of the reagents, and *L. bozemanii* was deemed to be the causative pathogen of the patient’s illness. He was started on oral azithromycin 12 mg/kg once daily with plans for a 2-week follow-up assessment, at which time his symptoms were improving but with small amounts of ongoing drainage from the surgical sites. He continued azithromycin therapy for an additional 2 weeks (4 weeks total), at which point his symptoms had fully resolved. He was evaluated again after 3 months with no recurrence of symptoms (Fig. 1).

## DISCUSSION

Historically, diagnosis of Legionellosis via traditional culture-based detection methods has been challenging. While culture-based identification remains the gold standard for diagnosis, this fastidious group of organisms requires plating on buffered charcoal yeast extract (BCYE) medium, where growth occurs slowly and can require up to 14 days delaying time to diagnosis (1). Because of this, BCYE medium is not commonly available in clinical laboratories, particularly at dedicated pediatric institutions such as at our facility. Plating on BCYE will only occur when specifically requested for cases with a high index of suspicion for pathogens, such as *Legionella spp.,* which were not initially suspected in this patient’s case. Immunoassays for detection of *Legionella* lipopolysaccharide antigen in the urine are widely available and can be useful in the diagnosis of Legionnaire’s disease; however, these tests are specific for *L. pneumophila* serogroup 1 and do not capture the presence of other pathogenic *Legionella* species (4). Similarly, serologic testing is not helpful for the diagnosis of acute Legionellosis; serum immunoglobulin (Ig)M levels are not predictive due to high rates of cross-reactivity with other Gram-negative pathogens, and while paired acute and convalescent IgG titers can identify recent infection, this is not useful to guide treatment decisions in the acute phase (1). 16S rRNA sequencing methods offer a novel approach for detecting all bacterial pathogens, including *Legionella* species, in tissue-based disease. PCR-based assays specifically targeting *Legionella* DNA are also increasingly available through major reference laboratories and may offer more rapid and sensitive *Legionella* detection in certain contexts compared to 16S rRNA sequencing (5). However, as *Legionella* species are not commonly thought to cause pediatric cervical lymphadenitis, dedicated Legionella PCR assays are not a part of routine diagnostic evaluation for culture-negative lymphadenitis; broader sequencing methods have the advantage of detecting causative pathogens not considered in the original differential diagnosis. Thus, the role of 16S rRNA sequencing and *Legionella* PCR testing in pediatric culture-negative lymphadenitis remains underexplored and warrants further investigation.

To date, only one study has evaluated the role of bacterial sequencing in pediatric culture-negative lymphadenitis (6). A retrospective case series from 1997 to 2007 at the University of Washington evaluated 60 otherwise-healthy children with persistent cervical lymphadenitis who underwent surgical intervention with negative routine cultures after 48 h of incubation. Samples were evaluated for pathogen identification via PCR methods, delayed culture, or histopathology. Of 49 positive diagnoses made by these methods, *Mycobacteria* were the most common organisms identified (37 cases), with 32 of the 37 identified by delayed AFB culture. The remainder of positive diagnoses were of *Bartonella henselae* (6) and *Legionella* species (6), diagnosed only using PCR and not identified by culture or histopathology. This was the first known report of persistent pediatric lymphadenitis caused by *Legionella* species, and the species identified were *L. pneumophila* and *L. micdadei*. Of the *Legionella* species which are known human pathogens, *L. pneumophila* and *L. micdadei* are frequently implicated in disease in immunocompetent hosts; however, in our case, the discovery of *Legionella bozemanii* as a pathogenic agent in an immunocompetent child is surprising.

*L. bozemanii* was first identified in 1959 and is most described as a causative agent of pneumonia in immunocompromised hosts, though in this demographic it has also been found to cause soft tissue and joint infections (3, 7). However, with PCR-based detection methods, there is increasing recognition of disease in immunocompetent adults, with isolated cases of pneumonia and endocarditis described (8, 9). To date, there is one case report of *L. bozemanii* causing refractory cervical lymphadenitis in a presumed immunocompetent adult after a dental procedure, and this pathogen was only identified via next-generation sequencing (10). The patient was treated with oral azithromycin 500 mg daily for 1 month and responded well to macrolide therapy. Given the variation in disease manifestations and immune status of hosts, there are no standardized guidelines for the treatment of *L. bozemanii infections*, though regimens most often include macrolide or fluoroquinolone agents which have proven efficacy against *Legionella* species (11). In the one other documented pediatric case of *L. bozemanii* which manifested as a lung abscess in a stem cell transplant recipient, the patient responded well to 5 months of oral macrolide therapy, which was prolonged due to immunocompromised status (12). Our patient’s response to 4 weeks of azithromycin is supportive of the use of shorter-course macrolide therapy for *L. bozemanii* lymphadenitis in otherwise healthy children; however, further cases are needed to guide standardized treatment recommendations. Additionally, it is worth noting that while our case is unique in identifying *L. bozemanii* infection in a previously healthy child, no further investigation was pursued to confirm his immunocompetency. As this was his first serious lifetime infection with no family history of recurrent infections or immunodeficiencies, there was no suspicion for an underlying immunodeficiency on the basis of this infection alone, which responded well to antimicrobial therapy. Furthermore, little is known regarding the effect of surgical approach on the patient’s clinical outcome; given the concern for nontuberculous mycobacteria in his initial presentation, excisional lymphadenectomy was recommended; however, intraoperatively, it was determined that curettage was the best strategy to effectively debride involved epithelium while limiting morbidity of the procedure. In the case series from the University of Washington, there was a small but significant difference in cure rate between incision and drainage and excisional lymphadenectomy (6). However, only one patient underwent incision and curettage, which was successful, though too small a sample size to determine significance. More research is needed to understand whether incision and curettage could be considered as a preferred surgical approach to cases of *Legionella* lymphadenitis.

### Conclusion

To our knowledge, this is the first documented pediatric case of *L. bozemanii* both as a cause of cervical lymphadenitis and as a pathogen in an immunocompetent child. Our patient’s treatment course was guided by molecular identification of this atypical bacteria, and he had excellent response to oral macrolide therapy where antibiotics directed against typical skin and soft tissue pathogens had previously failed. Given this and the challenges surrounding diagnosis, we posit that *L. bozemanii* is likely a rare though underappreciated cause of culture-negative lymphadenitis in immunocompetent children and that clinicians should lend consideration to the role of 16S rRNA sequencing in the diagnosis of culture-negative pediatric lymphadenitis.
